# Genetic Comparison of a Croatian Isolate and CEPH European Founders

**DOI:** 10.1002/gepi.20443

**Published:** 2010-02

**Authors:** Pau Navarro, Véronique Vitart, Caroline Hayward, Albert Tenesa, Lina Zgaga, Danica Juricic, Ozren Polasek, Nicholas D Hastie, Igor Rudan, Harry Campbell, Alan F Wright, Chris S Haley, Sara A Knott

**Affiliations:** 1 MRC Human Genetics Unit, Institute of Genetics and Molecular Medicine, Western General HospitalEdinburgh, UK; 2 Colon Cancer Genetics Group, University of Edinburgh, Western General HospitalEdinburgh, UK; 3 Andrija Stampar School of Public Health, Faculty of Medicine, University of ZagrebZagreb, Croatia; 4 Department of Internal Medicine, Zagreb University Hospital CenterZagreb, Croatia; 5 Community Health Sciences, University of EdinburghEdinburgh, UK; 6 Croatian Centre for Global Health, University of SplitSplit, Croatia; 7 Institute of Evolutionary Biology, University of Edinburgh, King's BuildingsEdinburgh, UK

**Keywords:** linkage disequilibrium, mapping, population isolate

## Abstract

Human isolates have been postulated as a good resource for the identification of QTL due to reduced genetic diversity and a more homogeneous environment. Isolates may also have increased linkage disequilibrium (LD) due to small effective population size and, either loss or increase in frequency of alleles that are rare in the general population from which they originate. Here we investigate the difference in allele and genotype frequencies, LD and homozygous tracts between an isolate—several villages from the island of Vis in Croatia—and an outbred population of European origin: the Hapmap CEPH founders. Using the HumanHap300 v1 Genotyping BeadChip, we show that our population does not differ greatly from the reference CEU outbred population despite having a slightly higher proportion of monomorphic loci, a slightly higher long-range LD, and a greater proportion of individuals with long homozygous tracts. We conclude that genotyping arrays should perform equally well in our isolate as in outbred European populations for disease mapping studies and that SNP–trait associations discovered in our well-characterized Croatian isolate should be valid in the general European population from which they descend. *Genet. Epidemiol*. 34: 140–145, 2010. © 2009 Wiley-Liss, Inc.

## INTRODUCTION

Human isolates have been postulated as a good resource for the identification of QTL due to reduced genetic diversity and a more homogeneous environment. Isolates may also have increased linkage disequilibrium (LD) due to small effective population size and, either loss or increase in frequency of alleles that are rare in the general population from which they originate [Wright et al., 1999].

Here we investigate the difference in allele and genotype frequencies, LD and homozygous tracts between an isolate—several villages from the island of Vis in Croatia—and an outbred population of European origin: the Hapmap CEPH founders (Utah residents with ancestry from northern and western Europe, CEU).

## MATERIAL AND METHODS

### POPULATION ISOLATE

Croatia has 15 Adriatic Sea islands with population greater than 1,000. The villages on the islands have unique population histories and preserved isolation from other villages and outside world through centuries. The village populations of these islands represent well-characterized genetic isolates [Bennett et al., 1983; Rudan et al., 1999; Rudan et al., 1992]. Komiza and Vis, on the island of Vis, have excellent church and census records that show evidence of very limited immigration from other populations and this is supported by the very high endogamy (calculated as the percentage of grandparents born in the same village as the participant) estimated for the villages: 91% for Komiza and 85% for Vis. Several rare autochthonous Mendelian diseases occur in these Adriatic islands [Bakija-Konsuo et al., 2002; Saftic et al., 2006] where at least four highly unusual rare genetic variants segregate [Barac et al., 2003; Borot et al., 1991; Tolk et al., 2001; Turcinov et al., 2000]. Each one of these findings is generally consistent with the hypothesis that all affected (carrier of a particular variant) chromosomes descend from a single founder.

### HUMAN SUBJECTS

60 founders from the CEPH European sample have been genotyped by the HapMap project.

For comparison we selected 60 unrelated (based upon their pedigrees obtained from church/parish records) and healthy individuals from our study population from the island of Vis, Croatia (CROATIA, for description see Vitart et al. [2008]).

### GENOTYPING

The Croatian samples were genotyped using the Illumina's Sentrix HumanHap300 Genotyping BeadChip (v1) comprising 317,503 SNPs. Genotypes for these same SNPs for the 60 CEPH founders were obtained from Illumina Inc.^1^

### STATISTICAL METHODS

In both populations, we excluded markers with less than 90% call rate in each population (9,075 in CROATIA, 71 in CEU, 9,088 in total) and markers on the sex chromosomes (9,173), leaving 299,242 SNPs. This set of SNPs was used to produce the results presented except when otherwise stated. We estimated the proportion of loci segregating in each population, the allele and genotype frequencies and the proportion of loci out of Hardy-Weinberg equilibrium (HWE). Data were analyzed using PLINK 1.04 [Purcell et al., 2007], R [R Development Core Team, 2008] and custom-made software.

#### Genetic distance between populations

Pairwise Fst statistics were calculated for all SNP markers segregating in both populations. A mean Fst was calculated for all markers and for the subset with minor allele frequency (MAF) 40.05 in both populations. We studied the sampling properties of Fst estimates by bootstrapping as suggested by Weir et al. [2005]. For a window of size 5 Mb (2.5 Mb to each side of each available SNP), we used 1,000 bootstrap samples to obtain the distribution of Fst estimates for that window, and obtained the mean Fst (Fstb) and 95% confidence intervals.

#### Linkage disequilibrium

LD was estimated as *r*^2^ and *D'*, for all segregating pairs of SNPs less than 10 Mb apart, and for the subset of these SNPs that were in HWE (*P*-value ≥0.01) and with MAFs ≥0.05 in both populations.

#### Homozygous tracts

Homozygous tracts over 200 kb in length were recorded by counting the number of consecutive homozygous SNPs including monomorphic SNPs. We allowed one heterozygous (i.e. one potential genotyping error) and one missing SNP genotype per 200 kb segment around each analyzed SNP, and recorded the positions of the start and end SNP for each tract. Tracts with an average density of less than one SNP per 50 kb or with less than 10 SNPs in total were excluded to avoid regions of low SNP coverage such as the centromeric regions. This analysis was implemented in PLINK 1.04, using a sliding window of 200 kb.

## RESULTS

The average inter-marker distance for adjacent marker pairs was 9,285 bp (range 1–22072916) (Figure S1). Table I summarizes the results on allele frequency in each population. In total 170 SNPs were monomorphic in both populations. The average MAF was similar at 0.25 for CROATIA and 0.26 for CEU; however, the distributions of allele frequencies differed. CROATIA exhibited an excess of loci in the 0–0.05 MAF range compared to CEU, but the trend was the opposite for loci with MAFs of 0.05–0.15. For MAFs of 0.15–0.5 the populations were very similar (Figure S2). Mean heterozygosity was also similar for the populations (34.17% (range 0–78.33%) for CROATIA and 34.98% (0–71.67%) for CEU). CROATIA had an excess of loci with lower heterozygosity (range 0–10%) compared to CEU but CEU had an excess of loci in the range 10–20%, with the rest of the distribution being similar (Figure S3).

**TABLE I:** Count (percentage) of monomorphic loci or with MAFs lower than 5% or between 5 and 10%

	MAF = 0	0<MAF≤5%	5%<MAF≤10%
CROATIA	813 (0.27%)	17272 (5.77%)	32727 (10.94%)
CEU	210 (0.07%)	7509 (2.50%)	39852 (13.32%)

We used the exact test for HWE described in Wigginton et al. [2005]. 3.41% of loci showed P-values o0.05 in CROATIA and 2.77% in CEU when only loci with MAFs 40.05 in both populations were used (for all loci, these figures were 3.25 and 2.64%, respectively), so no more loci were found to be out of HWE in either population than expected by chance.

### GENETIC DISTANCE BETWEEN POPULATIONS

Average Fst for these populations was 0.014 both when loci polymorphic in both populations were tested and when only loci with MAF 40.05 in both populations were used, indicating very little differentiation [S. Wright, 1978]. Overall the populations are quite similar, but several loci show high Fst values (Figure S4). A group of markers with Fst 40.25 is located on chromosome 2; it spans around 1.8 Mb and includes SNPs in the Lactase (LCT) gene. Results were similar when using all polymorphic loci or using only loci with MAF 40.05 in both populations (Figure S5). Weir et al. [2005] suggest that values of Fstb greater than the chromosome average plus three Fstb standard deviations reflect ‘^‘^truly exceptional differences^’^’ between populations. Again, the region around the LCT locus is highlighted by this method, and so are additional regions on chromosomes 3, 6 and 8, although less clearly.

### LINKAGE DISEQUILIBRIUM

**Fig. 1:**
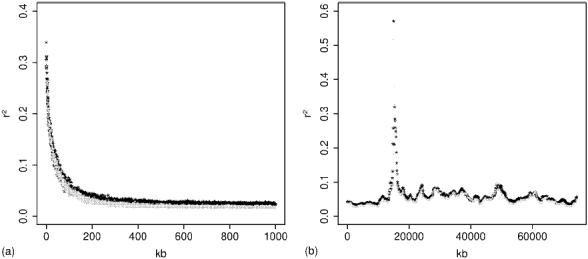
(a) Average *r*^2^ plotted against inter-SNP distance for chromosome 18 for CROATIA (black) and CEU (gray). (b) Distribution of *r*^2^ along chromosome 18 for CROATIA (black) and CEU (gray). Average *r*^2^ plotted for markers less than 500 kb apart, in sliding windows of 1.7 Mb, with a 1.6 Mb overlap.

Table II summarizes the results on LD for SNPs less than 10 Mb apart, in HWE (*P*-value ≥0.01) and with MAFs ≥0.05 in both populations. The proportions of marker-pairs either in perfect or “useful” LD are slightly higher for CROATIA than for CEU. Figure 1(a) shows plots of LD decay (average *r*^2^ for a given inter-marker distance, with markers distances grouped in 250 bp bins) with distance (up to 1 Mb) for chromosome 18 (gene poor). For this and the other autosomes (data not shown), CROATIA exhibits slightly higher *r*^2^ than CEU, and that is more evident for distances greater than 200 kb, where both populations seem to reach an “equilibrium long-range LD.” Figure 1(b) shows the moving average of *r*^2^ along chromosome 18. Again, consistently CROATIA shows higher *r*^2^ than CEU, for this chromosome but also for the remaining autosomes (data not shown). In supplementary materials, we have included the same figures for chromosome 19 (gene rich) (Figure S5).

**TABLE II:** Percentage of autosomal SNP pairs^a^ showing no evidence of recombination (*D*′ = 1), perfect LD (*r*^2^ = 1), or where useful LD is observed (*r*^2^≥0.8)

	*D*′ = 1		*r*^2^ = 1		*r*^2^≥0.8	
Inter-SNP distance (kb)	CROATIA	CEU	CROATIA	CEU	CROATIA	CEU
≤10	62.85	60.79	3.86	3.54	7.61	6.08
10–20	43.76	41.04	1.57	1.38	4.02	3.01
20–50	29.57	26.87	0.66	0.55	1.95	1.42
50–100	19.01	16.46	0.20	0.16	0.69	0.50
100–200	13.27	10.96	0.05	0.04	0.20	0.15
200–500	10.10	7.91	0.01	0.01	0.04	0.03
500–1,000	8.95	6.85	0.00	0.00	0.00	0.00
1,000–2,000	8.42	6.59	0.00	0.00	0.00	0.00
2,000–5,000	7.92	6.53	0.00	0.00	0.00	0.00
5,000–10,000	7.53	6.53	0.00	0.00	0.00	0.00

a Restricted to SNPs with minor allele frequency ≥0.05 and HWE *P*-value≥0.01 in both samples (*n* = 281,216).

### HOMOZYGOUS TRACTS

For homozygous runs greater than 200 kb, the average total homozygous tract length per individual was 145,191.8 kb for CROATIA and 122,790.6 kb for CEU. We grouped homozygous tracts in seven bins of increasing size, 200–500 kb, 500–1 Mb, 1–2 Mb, 2–5 Mb, 5–10 Mb, 10–20 Mb and over 20 Mb. CEU individuals showed an excess of shorter tracts when compared to CROATIA individuals, but the opposite trend was true for longer tracts (2Mb and over). Figures S6 and S7 give more details on the tract length and number distributions. In total, 13 individuals had individual tracts longer than 20 Mb. 12 of these individuals belong to CROATIA, whereas only one belongs to CEU. We compared locations of tracts longer than 2 Mb, that are found more often in CROATIA than in CEU, and observed that, in general, tracts found in CEU are also found in CROATIA (Figure S8).

## DISCUSSION

The aim of this study was to characterize an isolated population from the Dalmatian Island of Vis in Croatia in terms of allele frequencies, homozygosity and LD, and compare it to the CEU Hapmap sample. Our analyses revealed that although both populations show very similar average MAF, the Croatian isolate has a larger proportion of monomorphic loci than CEU, and a larger proportion of markers in the [0–0.05] MAF range. This trend is reversed for loci with MAFs in [0.05–0.15] and, for MAFs in [0.15–0.5] both populations are very similar. The Human-Hap300 v1 Genotyping BeadChip was designed based on CEU samples, and SNPs were selected to be relatively common (with allele frequencies >0.05) in that population and to exhibit low pairwise LD between each other. This could partly explain why CEU shows a deficit of SNPs with low allele frequencies when compared to CROATIA and a similar trend may have been observed if a different population to our study population had been compared to CEU, using the same panel of SNPs. It could also explain the relative enrichment of the CEU sample for SNPs with MAFs in [0.05–0.15]. Nonetheless, our findings are also consistent with a higher level of inbreeding (and low effective population size) of the Dalmatian isolate. We could hypothesize that a small number of population founders together with isolation (and drift) are responsible for a higher frequency of monomorphic loci in CROATIA when compared to CEU. We could further hypothesize that these same phenomena may potentially have caused some rare SNPs present in the general European population to be lost or conversely increase in frequency in the isolate. The total proportion of markers with MAF <0.10 in both populations studied is slightly above the range reported by Service et al. [2006] for a range of 11 population isolates. Their study used a set of around 2500 SNPs located on chromosome 22, and that might account for the difference observed. Average heterozygosity was also very similar between CROATIA and CEU, and also similar to that reported by Service et al. [2006] for their populations. Again, looking at the distribution of heterozygosity, we observe differences between CROATIA and CEU, with the former showing an excess of SNPs with lower heterozygosity with respect to the later, consistent with the differences in the distribution of allele frequencies. When testing for HWE, we did not observe more loci in disequilibrium than expected by chance, and that probably reflects the good quality of the genotyping. We proceeded to compare individual SNP allele frequencies and overall both populations were very similar, with the exception of few groups of SNPs that had Fst values >0.15. Among these loci is the LCT gene, which is known to have different allele frequencies across Europe [Bersaglieri et al., 2004; Burton et al., 2007]. We did not find reports describing differences for the remaining loci, which consist mostly of SNPs grouped within the same regions—rather than individual SNPs—and cover from few tens of kb to in excess of 1 Mb (for chromosome 6). Differences at some of these loci could be the result of (and are consistent with) the population having been founded by few individuals and/or of genetic drift.

We estimated LD between pairs of loci located less than 10 Mb apart for both CROATIA and CEU using the same sets of markers, both using all loci with a call rate > 90% in both populations or excluding loci with MAF <0.05 and HWE *P*-value >0.01. Summary results for these sets of markers did not differ significantly within populations. We have presented in Table II the proportions of marker pairs in perfect LD or “useful” LD, and these are slightly higher for CROATIA. We have also shown that consistently, CROATIA exhibits slightly higher *r*^2^ than CEU and that for distances greater than 200 kb, both populations reach what we call an “equilibrium long-range LD,” that is also slightly higher for CROATIA. Higher long-range LD could reflect that the population may have undergone a relatively recent bottleneck [Tenesa et al., 2007] and therefore would exhibit reduced variation (i.e. more monomorphic loci and lower heterogeneity). We used a genotyping array that has been designed to avoid genotyping markers in very high LD with each other. To explore how SNP ascertainment influenced our LD results, we downloaded the *r*^2^ estimates for chromosome 22 for CEU from www.hapmap.org (Phase II data, pairwise *r*^2^ estimates for SNPs up to 200 kb apart), and summarized these data as detailed for Table II (Table SI). We observed that results for our set of markers show consistently a lower proportion of markers with high *r*^2^ than the whole HapMap data set for all ranges of distances, which is consistent with the SNPs having been chosen to avoid high LD among them.

We scored homozygous tract length for each individual, and we show that both the average total tract length and the average count of tracts longer than 2 Mb is greater in CROATIA than in CEU, but this trend is reversed for shorter tracts, probably because longer tracts are broken down given the difference in number of monomorphic SNPs and SNPs with lower allele frequencies between the two populations. Gibson et al. [2006] showed that “long (over 1 Mb) homozygous tracts are relatively common even in the unrelated individuals from the outbred populations represented in the HapMap samples” and that they are usually located in regions of low recombination rate. They also claim that very long tracts of homozygosity, particularly if not associated with regions of low recombination, are likely to be a signature of recent inbreeding. 13 individuals in our study display very long (over 20 Mb) tracts of homozygous SNPs. Only one of these individuals (NA12874) is from the reference CEU outbred population. This individual has already been reported as having a particularly long tract and a higher total tract length when compared to the other CEU samples by Gibson et al. [2006]. They suggest that his parents are likely to share a recent common ancestor. All 12 Vis individuals displaying very long tracts also have higher than average total tract length and have both their parents born in the same village on the island of Vis (either Komiza, Vis, Okljuèna, Podhumlje or Podšpilje) except for one (for whom parental origin is unknown) so these individuals are probably the offspring of somehow related ancestors.

In all, using the HumanHap300 v1 Genotyping Bead-Chip, we have shown that our population does not differ greatly from the reference CEU outbred population, but has a slightly higher proportion of monomorphic loci, a slightly higher long-range LD, and a greater proportion of individuals with long homozygous tracts. These are consistent with genetic drift and high levels of endogamy, and with the demographic history of the isolate described by Vitart et al. [2006]. We can extrapolate that the trends we observe for genotyped loci will remain for untyped loci, and therefore conclude that genotyping arrays should perform equally well in our isolate than in outbred European populations for disease mapping studies. Furthermore, and as highlighted as well by Thompson et al. [2009] and Van Hout et al. [2009], susceptibility alleles should be the same in the isolates as in outbred European populations, so any findings made in those more homogeneous (in terms of environment) and well-characterized populations should be valid in the general European population from which they descend.

## WEB RESOURCES

HapMap data: http://www.hapmap.org/

## References

[b1] Bakija-Konsuo A, Basta-Juzbasic A, Rudan I, Situm M, Nardelli-Kovacic M, Levanat S, Fischer J, Hohl D, Loncaric D, Seiwert S, Campbell H (2002). Mal de Meleda: genetic haplotype analysis and clinicopathological findings in cases originating from the island of Mljet (Meleda), Croatia. Dermatology.

[b2] Barac L, Pericic M, Klaric IM, Rootsi S, Janicijevic B, Kivisild T, Parik J, Rudan I, Villems R, Rudan P (2003). Y chromosomal heritage of Croatian population and its island isolates. Eur J Hum Genet.

[b3] Bennett LA, Angel JL, Roberts DF, Rudan P (1983). Joint study of biological and cultural variation in Dalmatian village populations—project description. Coll Antropol.

[b4] Bersaglieri T, Sabeti PC, Patterson N, Vanderploeg T, Schaffner SF, Drake JA, Rhodes M, Reich DE, Hirschhorn JN (2004). Genetic signatures of strong recent positive selection at the lactase gene. Am J Hum Genet.

[b5] Borot N, Arnaud J, Rudan P, Chaventre A, Sevin J (1991). Phosphoglucomutase-1 subtypes in 2 populations in Adriatic Islands—presence of Pgm1-Star-W3 (Pgm1-Star-7+) allele. Hum Hered.

[b6] Burton PR, Clayton DG, Cardon LR, Craddock N, Deloukas P, Duncanson A, Kwiatkowski DP, McCarthy MI, Ouwehand WH, Samani NJ, Todd JA, Donnelly P, Barrett JC, Davison D, Easton D, Evans D, Leung HT, Marchini JL, Morris AP, Spencer CCA, Tobin MD, Attwood AP, Boorman JP, Cant B, Everson U, Hussey JM, Jolley JD, Knight AS, Koch K, Meech E, Nutland S, Prowse CV, Stevens HE, Taylor NC, Walters GR, Walker NM, Watkins NA, Winzer T, Jones RW, McArdle WL, Ring SM, Strachan DP, Pembrey M, Breen G, St Clair D, Caesar S, Gordon-Smith K, Jones L, Fraser C, Green EK, Grozeva D, Hamshere ML, Holmans PA, Jones IR, Kirov G, Moskvina V, Nikolov I, O'Donovan MC, Owen MJ, Collier DA, Elkin A, Farmer A, Williamson R, McGuffin P, Young AH, Ferrier IN, Ball SG, Balmforth AJ, Barrett JH, Bishop DT, Iles MM, Maqbool A, Yuldasheva N, Hall AS, Braund PS, Dixon RJ, Mangino M, Stevens S, Thompson JR, Bredin F, Tremelling M, Parkes M, Drummond H, Lees CW, Nimmo ER, Satsangi J, Fisher SA, Forbes A, Lewis CM, Onnie CM, Prescott NJ, Sanderson J, Mathew CG, Barbour J, Mohiuddin MK, Todhunter CE, Mansfield JC, Ahmad T, Cummings FR, Jewell DP, Webster J, Brown MJ, Lathrop GM, Connell J, Dominiczak A, Marcano CAB, Burke B, Dobson R, Gungadoo J, Lee KL, Munroe PB, Newhouse SJ, Onipinla A, Wallace C, Xue MZ, Caulfield M, Farrall M, Barton A, Bruce IN, Donovan H, Eyre S, Gilbert PD, Hider SL, Hinks AM, John SL, Potter C, Silman AJ, Symmons DPM, Thomson W, Worthington J, Dunger DB, Widmer B, Frayling TM, Freathy RM, Lango H, Perry JRB, Shields BM, Weedon MN, Hattersley AT, Elliott KS, Groves CJ, Lindgren CM, Rayner NW, Timpson NJ, Zeggini E, Newport M, Sirugo G, Lyons E, Vannberg F, Brown MA, Franklyn JA, Heward JM, Simmonds MJ, Hill AVS, Bradbury LA, Farrar C, Pointon JJ, Wordsmith P, Gough SCL, Seal S, Stratton MR, Rahman N, Ban M, Goris A, Sawcer SJ, Compston A, Conway D, Jallow M, Bumpstead SJ, Chaney A, Downes K, Ghori MJR, Gwilliam R, Inouye M, Keniry A, King E, McGinnis R, Potter S, Ravindrarajah R, Whittaker P, Withers D, Easton D, Pereira-Gale J, Hallgrimsdottir IB, Howie BN, Su Z, Teo YY, Vukcevic D, Bentley D, Caulfield M, Mathew CG, Worthington J (2007). Genome-wide association study of 14,000 cases of seven common diseases and 3,000 shared controls. Nature.

[b7] R Development Core Team (2008). R: A Language and Environment for Statistical Computing.

[b8] Gibson J, Morton NE, Collins A (2006). Extended tracts of homozygosity in outbred human populations. Hum Mol Genet.

[b9] Purcell S, Neale B, Todd-Brown K, Thomas L, Ferreira MAR, Bender D, Maller J, Sklar P, de Bakker PIW, Daly MJ, Sham PC (2007). PLINK: a tool set for whole-genome association and population-based linkage analyses. Am J Hum Genet.

[b10] Rudan I, Campbell H, Rudan P (1999). Genetic epidemiological studies of Eastern Adriatic island isolates, Croatia: objectives and strategies. CollAntropol.

[b11] Rudan P, Sujoldzic A, Simic D, Bennett LA, Roberts DF (1992). Population-structure in the Eastern Adriatic—the influence of historical processes, migration patterns, isolation and ecological pressures, and their interaction. Isolation Migration Health.

[b12] Saftic V, Rudan D, Zgaga L (2006). Mendelian diseases and conditions in Croatian island populations: historic records and new insights. Croat Med J.

[b13] Service S, DeYoung J, Karayiorgou M, Roos JL, Pretorious H, Bedoya G, Ospina J, Ruiz-Linares A, Macedo A, Palha JA, Heutink P, Aulchenko Y, Oostra B, van Duijn C, Jarvelin MR, Varilo T, Peddle L, Rahman P, Piras G, Monne M, Murray S, Galver L, Peltonen L, Sabatti C, Collins A, Freimer N (2006). Magnitude and distribution of linkage disequilibrium in population isolates and implications for genome-wide association studies. Nat Genet.

[b14] Tenesa A, Navarro P, Hayes BJ, Duffy DL, Clarke GM, Goddard ME, Visscher PM (2007). Recent human effective population size estimated from linkage disequilibrium. Genome Res.

[b15] Thompson EE, Sun Y, Nicolae D, Ober C (2009). Shades of gray: a comparison of linkage disequilibrium between Hutterites and Europeans. Genet Epidemiol.

[b16] Tolk HV, Barac L, Pericic M, Klaric IM, Janicijevic B, Campbell H, Rudan I, Kivisild T, Villems R, Rudan P (2001). The evidence of mtDNA haplogroup F in a European population and its ethnohistoric implications. Eur J Hum Genet.

[b17] Turcinov D, Krishnamoorthy R, Janicijevic B, Markovic I, Mustac M, Lapoumeroulie C, Chaventre A, Rudan P (2000). Anthropogenetical analysis of abnormal human alpha-globin gene cluster arrangement on chromosome 16. Coll Antropol.

[b18] Van Hout CV, Levin AM, Rampersaud E, Shen H, O'Connell J, Mitchell BD, Shuldiner AR, Douglas JA (2009). Extent and distribution of linkage disequilibrium in the old order Amish. Genet Epidemiol.

[b19] Vitart V, Biloglav Z, Hayward C, Janicijevic B, Smolej-Narancic N, Barac L, Pericic M, Klaric IM, Skaric-Juric T, Barbalic M, Polasek O, Kolcic I, Carothers A, Rudan P, Hastie N, Wright A, Campbell H, Rudan I (2006). 3000 years of solitude: extreme differentiation in the island isolates of Dalmatia, Croatia. Eur J Hum Genet.

[b20] Vitart V, Rudan I, Hayward C, Gray NK, Floyd J, Palmer CN, Knott SA, Kolcic I, Polasek O, Graessler J, Wilson JF, Marinaki A, Riches PL, Shu X, Janicijevic B, Smolej-Narancic N, Gorgoni B, Morgan J, Campbell S, Biloglav Z, Barac-Lauc L, Pericic M, Klaric IM, Zgaga L, Skaric-Juric T, Wild SH, Richardson WA, Hohenstein P, Kimber CH, Tenesa A, Donnelly LA, Fairbanks LD, Aringer M, McKeigue PM, Ralston SH, Morris AD, Rudan P, Hastie ND, Campbell H, Wright AF (2008). SLC2A9 is a newly identified urate transporter influencing serum urate concentration, urate excretion and gout. Nat Genet.

[b21] Weir BS, Cardon LR, Anderson AD, Nielsen DM, Hill WG (2005). Measures of human population structure show heterogeneity among genomic regions. Genome Res.

[b22] Wigginton JE, Cutler DJ, Abecasis GR (2005). A note on exact tests of Hardy-Weinberg equilibrium. Am J Hum Genet.

[b23] Wright AF, Carothers AD, Pirastu M (1999). Population choice in mapping genes for complex diseases. Nat Genet.

[b24] Wright S (1978). Evolution and the Genetics of Populations. Variability Within and Among Natural Populations.

