# Supplementary material for: Genetic Comparison of a Croatian Isolate and CEPH European Founders
Source: Genet Epidemiol. 2010 Feb;34(2):140–5. doi: 10.1002/gepi.20443 (PMC2896723; doi:10.1002/gepi.20443)

Supporting Information for "Genetic Comparison of a Croatian Isolate and CEPH European Founders"

COMPARISON OF LD ACROSS CHROMOSOMES: CHROMOSOME 18 VS 19

Figure S5a shows plots of LD decay (average r^2 for a given inter-marker distance, with markers grouped in 250 bp bins) with distance (up to 1 Mb) for chromosomes 18 (gene poor) and 19 (gene rich) for CROATIA and CEU. For both populations, chromosome 18 exhibits slightly lower average r^2 at very short distances (<1 kb), but also a lower rate of decay than chromosome 19, which results in chromosome 18 showing higher average r^2 for distances up to 250 kb. For both these chromosomes and the other autosomes (data not shown), CROATIA exhibits slightly higher r^2 than CEU, and that is more evident for distances greater than 200 kb, where both populations seem to reach an "equilibrium long-range LD". Figure S5b shows the moving average of r^2 along chromosomes 18 and 19. Again, consistently CROATIA shows higher r^2 than CEU, for these two chromosomes but also for the remaining autosomes (data not shown).

Fig. S1.Distribution of inter-marker distances for adjacent SNPs. Counts for inter-marker distances greater than 80 kb, have been grouped together at the rightmost bin of the distribution.

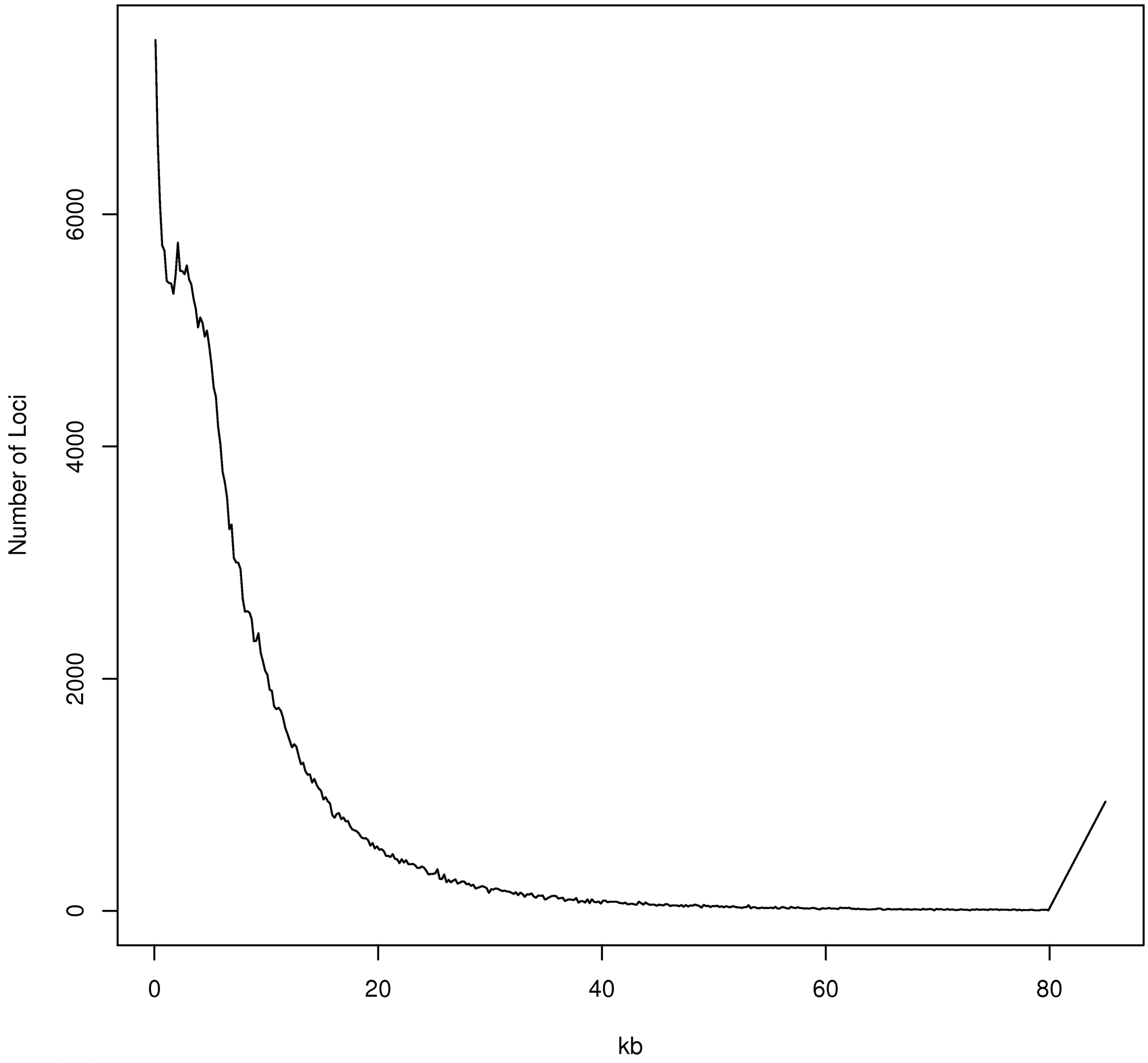

Fig. S2. Distribution of minor allele frequencies for CROATIA and CEU.

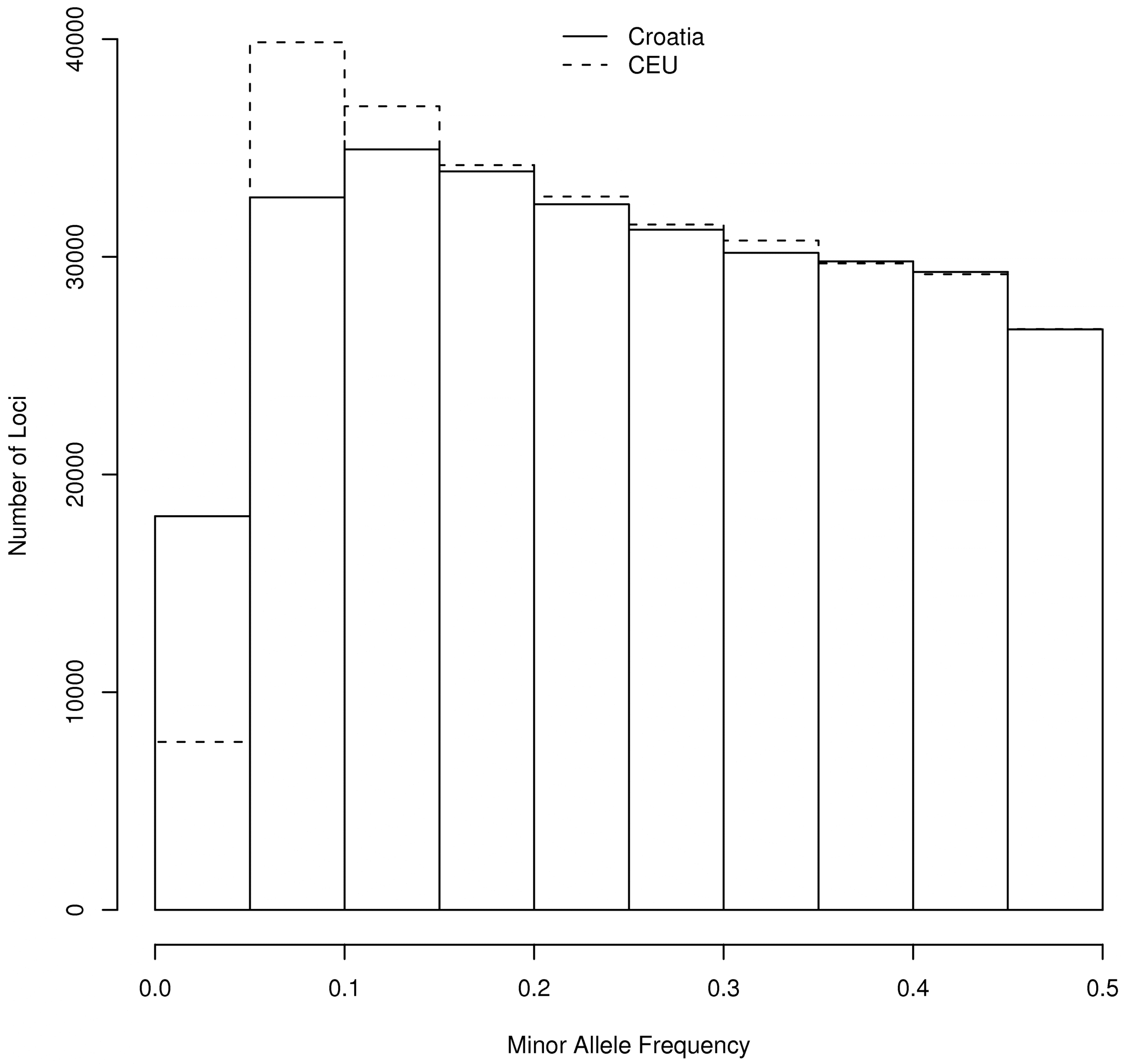

Fig. S3. Distribution of heterozygosity (number of loci with a given heterozygosity) for CROATIA and CEU.

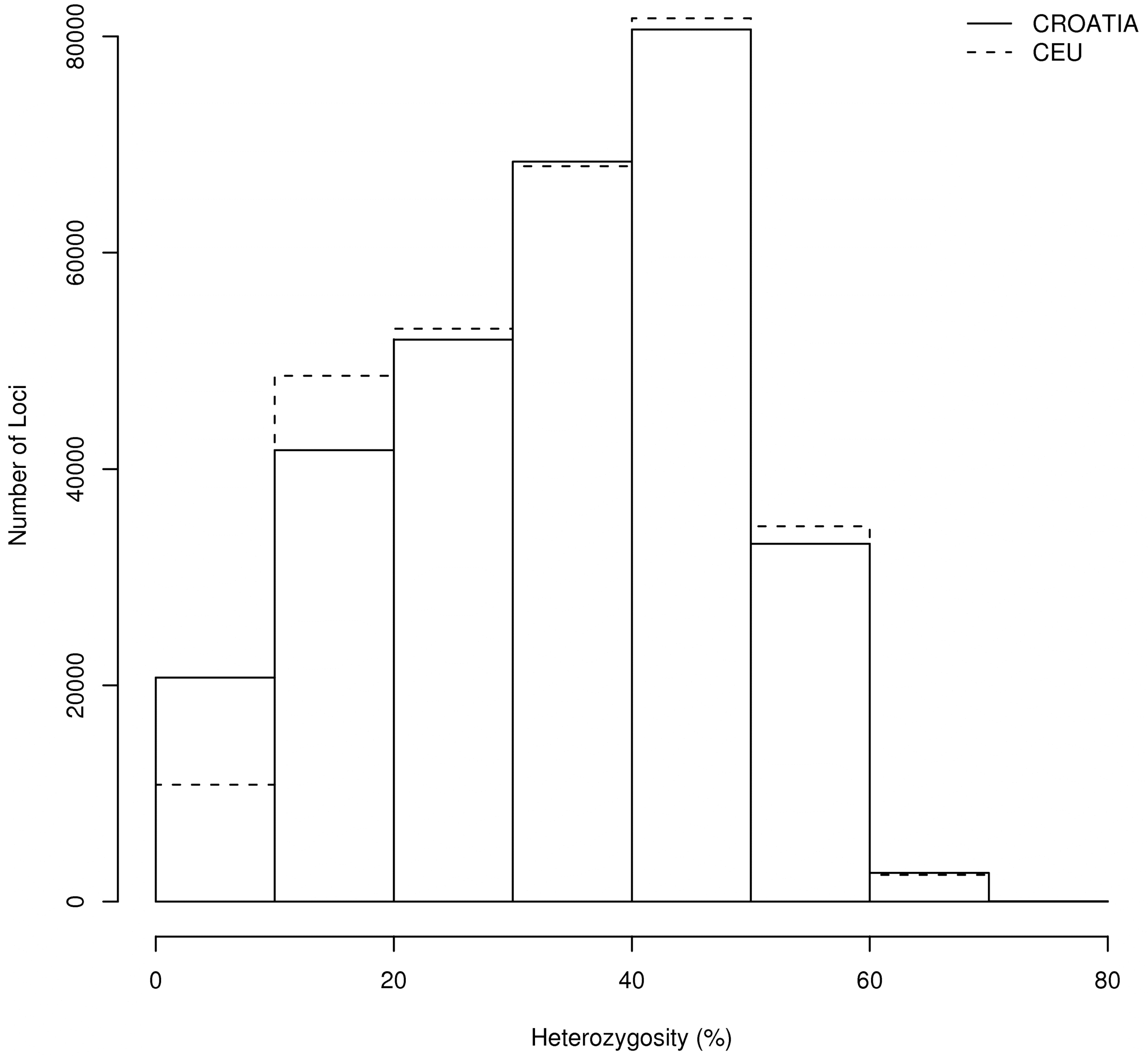

Fig. S4. Fst for CROATIA and CEU at each SNP polymorphic in both populations (black). In pink, Fst for SNPs with MAF>0.05 in both populations. In blue, a line marking the threshold for moderate differentiation and in red for great differentiation between the populations.

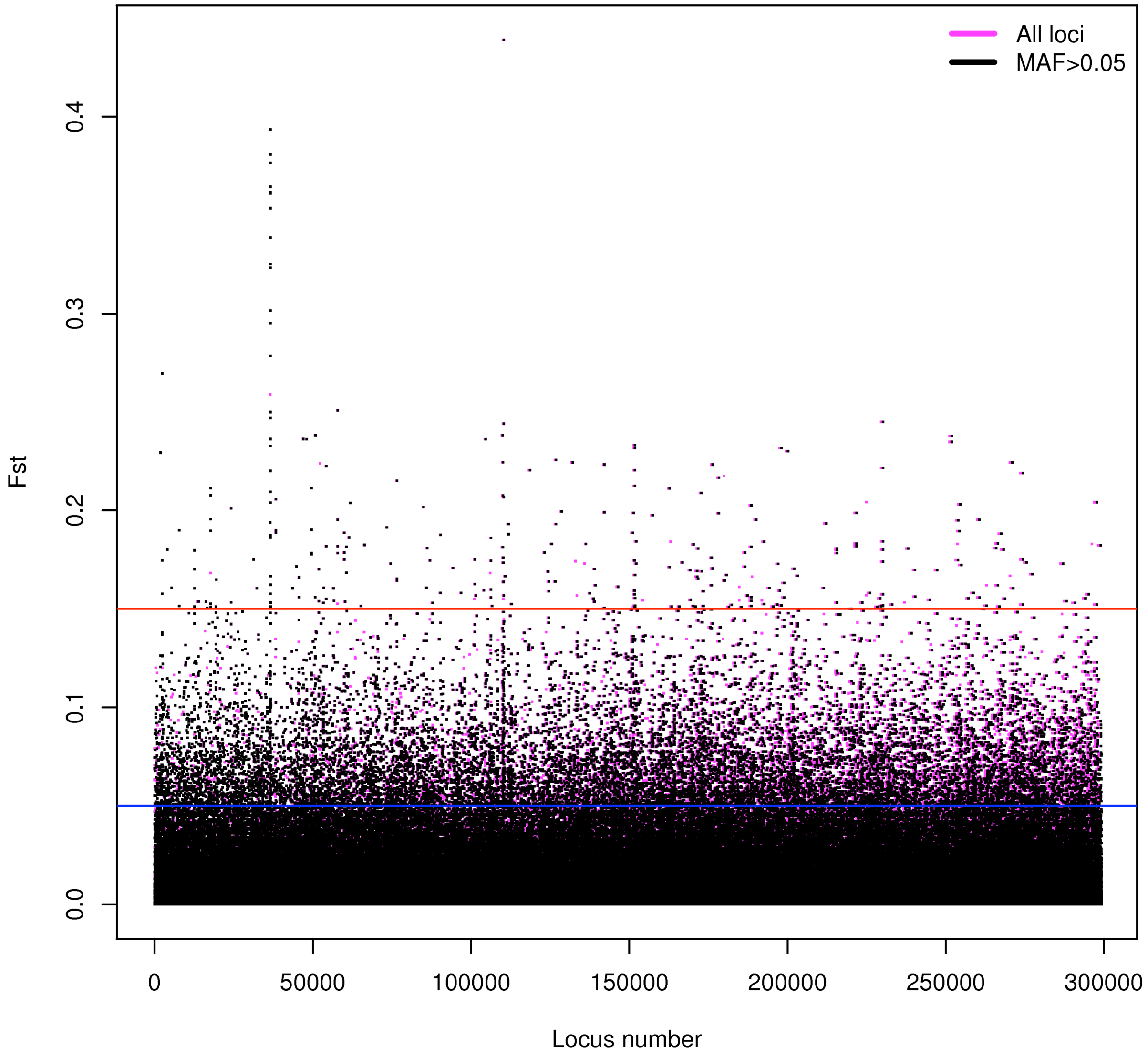

Fig. S5. (a) Average r^2 plotted against inter-SNP distance for chromosomes 18 and 19, for CROATIA (black) and CEU (gray). The red horizontal line represents r^2=0.025. (b) Distribution of r^2 along chromosomes 18 and 19, for CROATIA (black) and CEU (gray). Average r^2 plotted for markers less than 500 kb apart, in sliding windows of 1.7 Mb, with a 1.6 Mb overlap. The red horizontal line represents r^2=0.05.

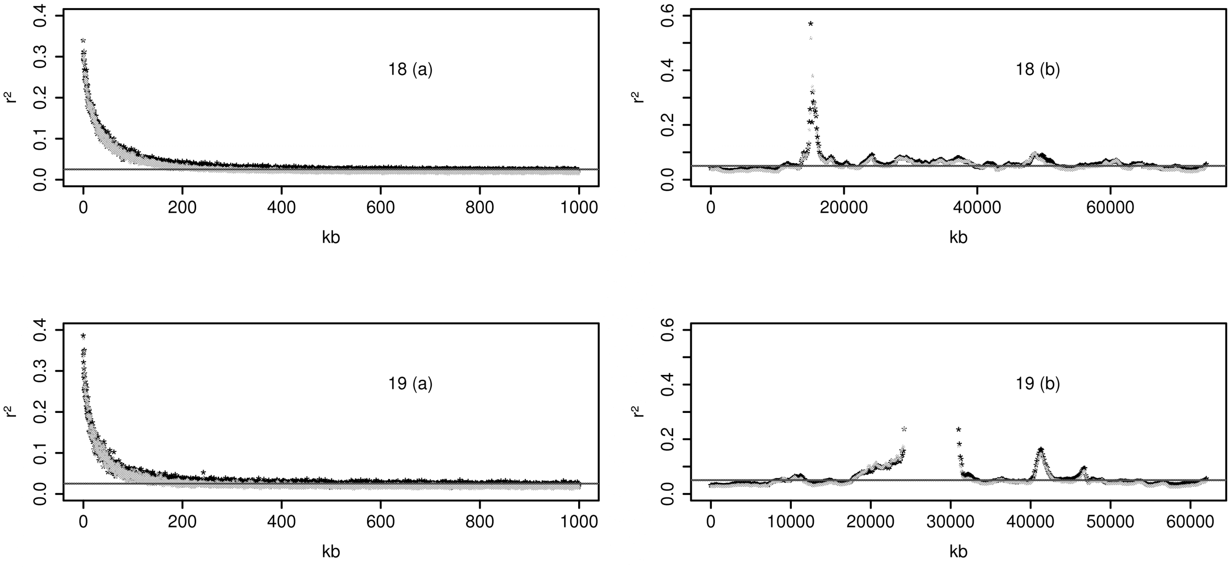

Fig. S6. Distribution of the total homozygous tract length (for tracts longer than 200kb) per individual for CROATIA and CEU.

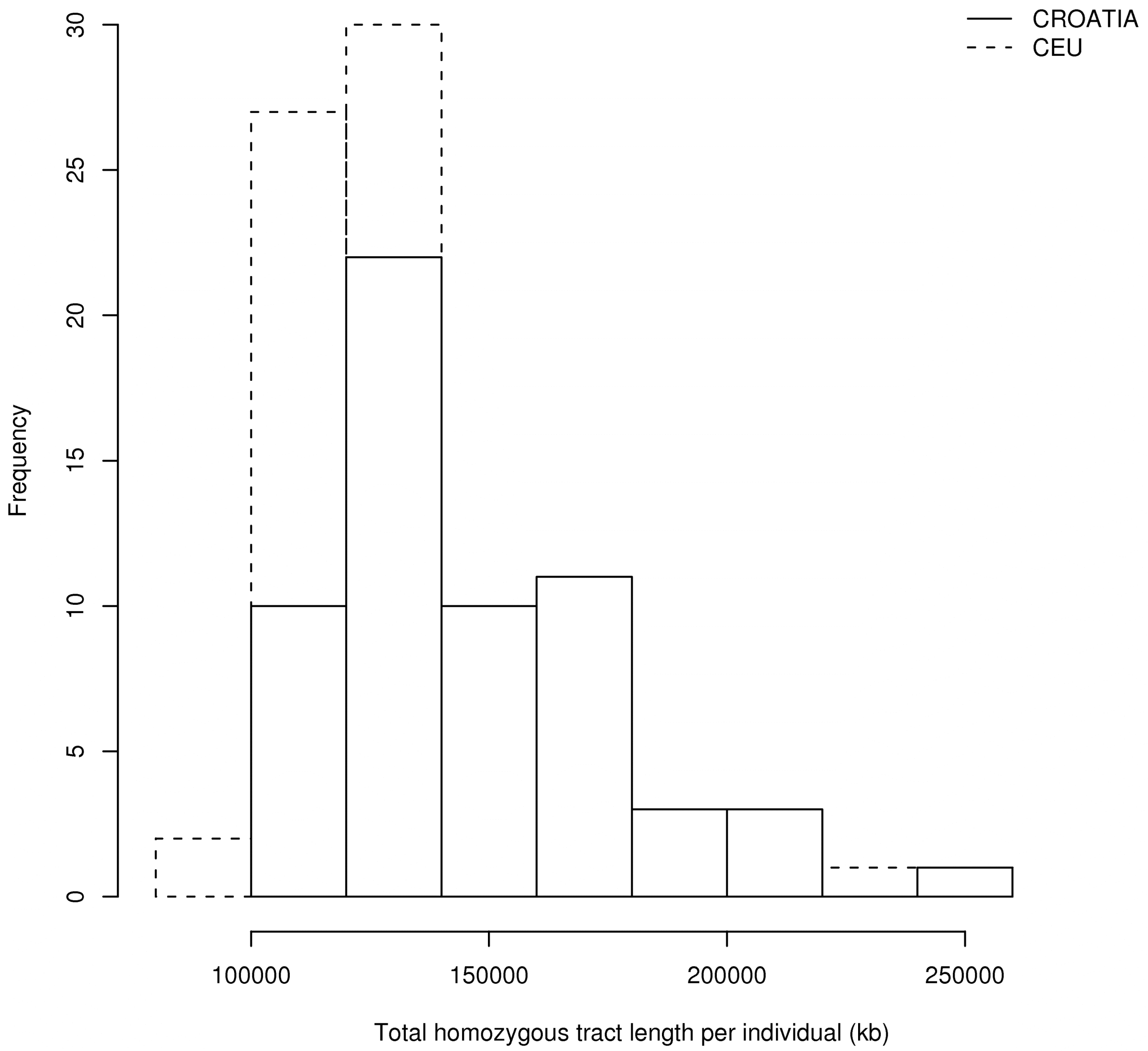

Fig. S7. Distribution of homozygous tracts in CROATIA and CEU. The average count per individual was calculated for 7 homozygous tract length categories; the leftmost figure shows the distribution over the whole range and the rightmost figure the distribution of tracts over 2Mb.

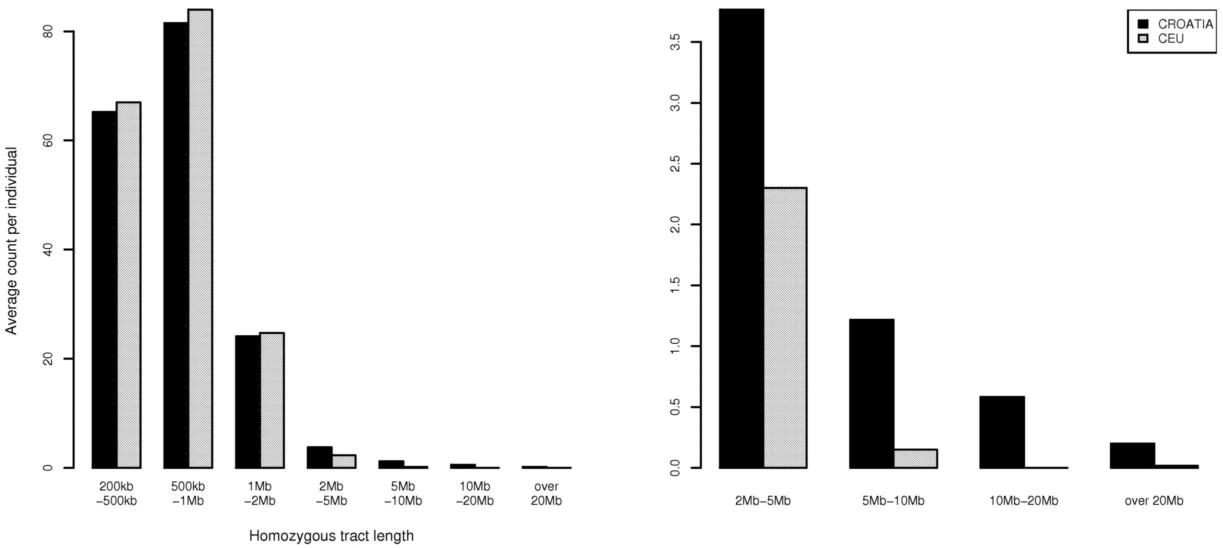

Fig. S8. Homozygous tracts longer than 2Mb plotted at their chromosomal location per individual. Individuals from CEU and CROATIA are plotted above and below the horizontal gray line respectively. Each individual's tracts are plotted on a line, and neighboring individuals are plotted in a different color for clarity. The red horizontal lines at the left hand side of the figure mark individuals with tracts longer than 20 Mb. Tracts longer than 2Mb are found more often in CROATIA than in CEU. The figure also shows which tracts are common to both populations. In general tracts found in CEU are also found in CROATIA.

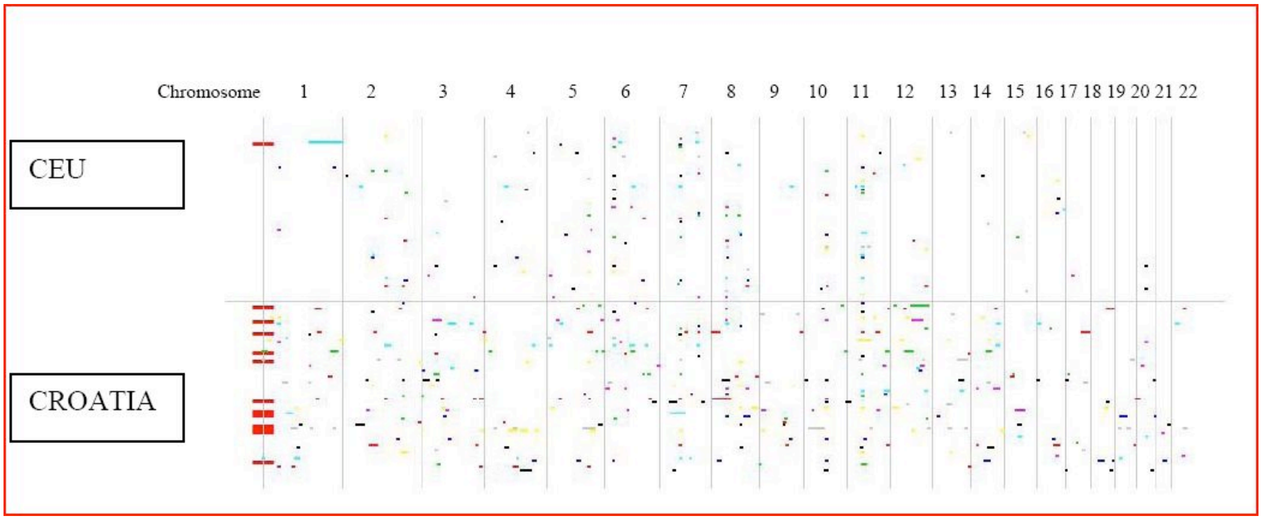

Supplement: Supplementary file 1 [file gepi0034-0140-SD1.pdf]
