# Supplementary material for: Genetic Comparison of a Croatian Isolate and CEPH European Founders
Source: Genet Epidemiol. 2010 Feb;34(2):140–5. doi: 10.1002/gepi.20443 (PMC2896723; doi:10.1002/gepi.20443)
Supplement: Supplementary file 2 [file gepi0034-0140-SD2.pdf]

TABLE SI. INFLUENCE OF SNP ASCERTAINMENT ON LD. Percentage and counts of SNP pairs from chromosome 22 showing perfect LD (r^2=1), or where useful LD is observed ($r^2 \geq 0.8$) for CEU, obtained either with the set of markers used in our study or the complete set of markers for chromosome 22 from the HapMap phase II data

Inter-SNP distance (kb)	Own data (#SNPs=4985)					HapMap Phase II data (#SNPs=35185)				
	Percentages		Counts			Percentages		Counts		
	r^2=1	$r^2 \geq 0.8$	Total	r^2=1	$r^2 \geq 0.8$	r^2=1	$r^2 \geq 0.8$	Total	r^2=1	$r^2 \geq 0.8$
≤10	2.82	5.38	9987	282	537	8.57	14.57	497563	42642	72494
10-20	0.66	1.96	9811	65	192	2.96	6.2	454934	13462	28222
20-50	0.28	0.71	28206	78	200	1.4	2.92	1315067	18389	38392
50-100	0.05	0.23	44664	24	103	0.51	1.13	2135300	10794	24097
100-200	0.02	0.07	85740	13	59	0.14	0.37	4146907	5862	15156
